# Bronchoscopic biopsies - a novel source for primary airway epithelial cells in respiratory research

**DOI:** 10.1186/s12931-024-03060-1

**Published:** 2024-12-24

**Authors:** Kimberly Barbet, Mona S. Schmitz, Dirk Westhölter, Markus Kamler, Stephan Rütten, Anja L. Thiebes, Barbara Sitek, Malte Bayer, Michaela Schedel, Sebastian Reuter, Kaid Darwiche, Anja E. Luengen, Christian Taube

**Affiliations:** 1 Department of Pulmonary Medicine, University Medical Center Essen, Ruhrlandklinik, Essen, Germany; 2 Department of Thoracic and Cardiovascular Surgery, University Medical Center Essen, Essen, Germany; 3 Institute of Pathology, Electron Microscopy Facility, RWTH Aachen University Hospital, Aachen, Germany; 4 Department of Biohybrid and Medical Textiles (BioTex), AME - Institute of Applied Medical Engineering, Helmholtz Institute, RWTH Aachen University, Aachen, Germany; 5 Medical Proteom-Center (MPC) Medical Faculty, Ruhr-University Bochum, Bochum, Germany; 6 Department of Anesthesiology, Intensive Care Medicine and Pain Therapy, University Hospital Knappschaftskrankenhaus, Bochum, Germany; 7 Department of Pulmonology, University Medical Center Essen, Essen, Germany; 8 Present address: Basic and Translational Lung Research, Departments of Pneumology, Mainz University Medical Center, Mainz, Germany; 9 Interventional Pulmonology, Department of Pulmonary Medicine, University Medical Center Essen, Ruhrlandklinik, Essen, Germany; 10 Department of Translational Pulmonology, Department of Pulmonary Medicine, University Medical Center Essen, Ruhrlandklinik, Essen, Germany

**Keywords:** Air-liquid interface, Primary airway epithelial cells, Forceps biopsy, Cryo biopsy, In vitro disease-model, 3R-principle

## Abstract

**Background:**

Using primary airway epithelial cells (AEC) is essential to mimic more closely different types and stages of lung disease in humans while reducing or even replacing animal experiments. Access to lung tissue remains limited because these samples are generally obtained from patients who undergo lung transplantation for end-stage lung disease or thoracic surgery for (mostly) lung cancer. We investigated whether forceps or cryo biopsies are a viable alternative source of AEC compared to the conventional technique.

**Methods:**

AECs were obtained ex vivo from healthy donor lung tissue using the conventional method and two biopsy procedures (forceps, cryo). The influence of the isolation method on the quality and function of AEC was investigated at different time-points during expansion and differentiation in air-liquid interface cultures. In addition, fully-differentiated AECs were stimulated with house dust mite extract (HDM) to allow functional analyses in an allergic in vitro model. Vitality or differentiation capacity were determined using flow cytometry, scanning electron microscope, periodic acid-Schiff reaction, immunofluorescence staining, and proteomics.

**Results:**

As anticipated, no significant differences between each of the sampling methods were detected for any of the measured outcomes. The proteome composition was comparable for each isolation method, while donor-dependent effects were observed. Treatment with HDM led to minor differences in mucociliary differentiation.

**Conclusions:**

Our findings confirmed the adequacy of these alternative approaches for attaining primary AECs, which can now expand the research for a broader range of lung diseases and for studies at an earlier stage not requiring lung surgery.

**Supplementary Information:**

The online version contains supplementary material available at 10.1186/s12931-024-03060-1.

## Introduction

The epithelium of the lung forms the first protective barrier against environmental factors. Is the epithelial barrier function disrupted, pathogens, pollutants or allergens can enter the body and cause lasting damage to the lung. The respiratory epithelium has therefore multiple key roles in the lung including the modulation of immune responses, each of which is needed for a healthy and efficient respiratory system. These are mediated by various epithelial cell types comprising of basal, goblet, ciliated, and club cells. Basal cells act as progenitor cells for the renewal of other epithelial cell types [1], while goblet cells secrete mucus to trap and eliminate inhaled particles and microorganisms [2]. Ciliated cells are responsible for moving mucus and trapped particles out of the airways through coordinated beating of their cilia [3], while club cells are involved in detoxification and epithelial regeneration [4] to ensure proper gas exchange of the lung. Epithelial function can be affected by various airway and lung diseases including chronic obstructive pulmonary disease (COPD), asthma, and cystic fibrosis. Patients with these diseases show squamous metaplasia, inflammatory cell infiltration, goblet cell hyperplasia, and mucus hypersecretion [5–7]. Furthermore, airway epithelial cells (AECs) are involved in innate immune responses and play a central role in orchestrating pulmonary inflammatory and immune reactions [8–10]. Although novel insights into the pathogenesis of these diseases have been achieved, there are still major gaps regarding the specific involvement of epithelial cells.

While animal models offer a way to characterize complex cellular interactions, the call for animal-free models is growing in society and the research community. Due to the lack of proper in vitro models, animal models often represent the method of choice to improve the limited understanding of the underlying mechanisms and the development of alternative therapy options in respiratory disease. However, adequate in vitro experiments using primary human cells are key to avoid animal experiments (replacement), to limit the number of animals (reduction), and their suffering to an absolute minimum (refinement). Overall, well-develop in vitro models may even reflect more closely human diseases.

In vitro cultures of primary human AECs deviate from standard submerged culture by cultivating cells in air-liquid interface (ALI). ALI-AECs are thus a valuable tool for investigating the general cell biology of the respiratory epithelium, studying epithelial infections, and modelling respiratory disease additionally in response to the exposure to various extrinsic factors or allergens [11, 12]. In allergic asthma, lung inflammation triggered by the clinically-relevant house dust mite (HDM) allergen causes allergic reactions in AECs leading to morphological changes, cell desquamation, and pro-inflammatory cytokine production [13]. In contrast to immortalised or tumour cell lines, primary cells have a limited life span but have the advantage of high differentiation capacity, especially when cultivated in ALI over several weeks [14]. Primary ALI-AECs therefore exhibit to date the most comparable phenotype to respiratory epithelium in vivo [11, 14, 15].

One of the major limitations of cultivating ALI-AECs is cell availability, especially from patients with early-stage lung disease. In several severe lung conditions, such as emphysema, cystic fibrosis, pulmonary fibrosis, sarcoidosis, pulmonary hypertension or other end-stage lung diseases, lung transplantation remains the only treatment option because all other therapies have failed [16]. The end-stage of each of these diseases can be well imaged in cell culture systems by isolating primary epithelial cells from diseased transplant tissue. In contrast, in vitro modelling of early stage-diseases or conditions that rarely lead to lung transplantation, such as asthma, remains challenging due to the limited availability of patient tissue. We therefore analyzed the potential of bronchoscopic tissue biopsies, a state-of-the-art technology in lung disease diagnosis, to obtain sufficient material for in vitro modelling of the respiratory tract. The use of biopsies during endoscopic procedures as a source of tracheal or bronchial epithelial cells has to date not been evaluated but can expand the spectrum of diseases to be analyzed using an autologous in vitro cell culture model.

## Methods

### Sample collection and primary AEC isolation of tissue obtained by conventional sampling method, forceps or cryo

For this study, tracheal tissue was obtained from healthy donors of lung transplants through the Department Clinic for Thoracic and Cardiovascular Surgery (University Hospital Essen). General selection criteria for lung transplant donors are listed in the Eurotransplant guidelines (https://www.eurotransplant.org/wp-content/uploads/2022/02/H1-Introduction-July-28-2016.pdf). Tissue was resected during the course of size adaptations of the donor lung before transplantation into the recipient. Three tissue sampling procedures were performed to evaluate the degree of comparability of the cultivation, differentiation, and functionality of primary AECs.

**Fig. 1:**
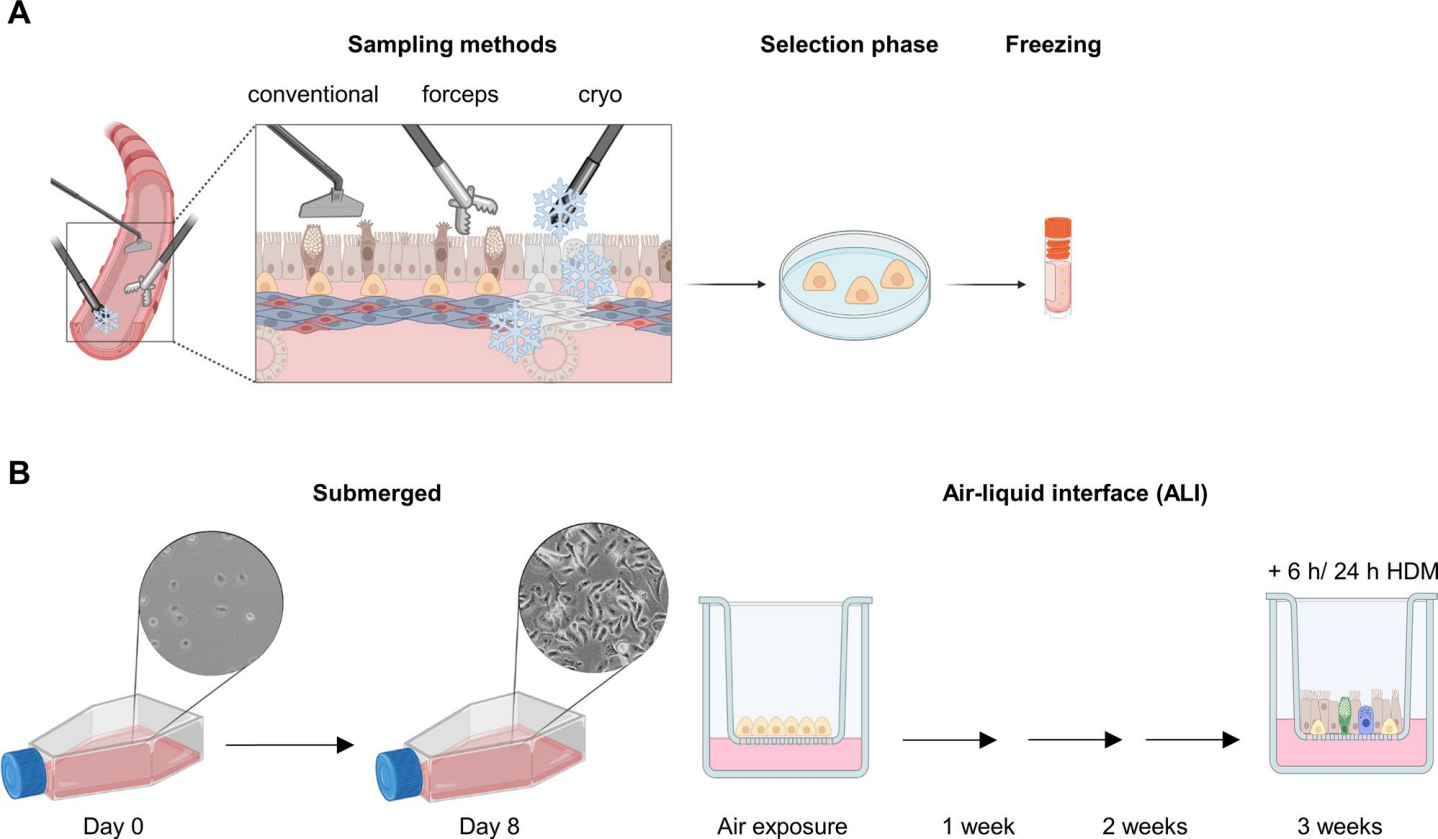
Sampling methods and cultivation of primary airway epithelial cells. **(A)** Tracheal tissue was resected in the course of size adaptations of donor lungs before transplantation. Three different sampling methods were used: conventional, forceps biopsy, and cryo biopsy. Epithelial cells were isolated and cultivated in specific media during the selection phase of basal cells. Basal cells were then frozen (400,000 cells/vial) and stored. **(B)** Isolated basal cells were expanded in submerged culture in T75 flasks for 8 days and seeded (40,000 cells) onto cell culture inserts. At a transepithelial electrical resistance value of 224 Ω/cm^2^, the apical medium was removed and cells were exposed to air. Cells were then cultivated for 3 weeks in air-liquid interface and harvested after 1, 2, or 3 weeks. After 3 weeks, some cells were treated with 10 µg/mL house dust mite extract (HDM) for 6–24 h to be used for functional analyzes. Figure created with Biorender

A common standardized protocol was used to isolate epithelial cells ex vivo after enzymatic digestion and mechanical isolation (referred to as “conventional method” [17] (Fig. 1a). Initially, fat and other tissue residues was removed from lung tissue before incubation in protease from *Streptomyces griseus* (182 µg/mL, Protease XIV, Sigma Aldrich, Burlington, United States) for 2 hours (h) at 37 °C. After incubation, the lung tissue was transferred to a sterile dish with 1x Dulbecco’s phosphate buffered saline (DPBS w/o calcium and magnesium, PAN-Biotech, Aidenbach, Germany) to stop the lung digestion. AECs were then scraped off the inner surface of the tracheal ring with a cell scraper and then with the blunt side of a scalpel. The cell suspension was centrifuged for 8 minutes (min) at 400 g at room temperature (RT). Subsequently, cells were washed with 10 mL 1x DPBS. A 6-well plate was coated with 1.5 mL/well of a mixture of fibronectin (5 µg/mL, Sigma Aldrich), PureCol (0.03 mg/mL, Advanced BioMatrix, Carlsbad, United States) and bovine serum albumin (BSA, 1 mg/mL, Sigma Aldrich) for 2 h at 37 °C. The cell suspension was centrifuged for 8 min (400 g, RT). Cells were then seeded in 1.5 mL media per well containing keratinocyte-serum-free media (KSFM media, Gibco, Waltham, United States) with epidermal growth factor (EGF, 2.5 µg, Gibco), bovine pituitary extract (BPE, 25 mg, Gibco), isoproterenol (1 µM, Sigma Aldrich), penicillin-streptomycin (P/S, 10 U/mL, Gibco), amphotericin B (2.5 µg/mL, PAN-Biotech) and ciprofloxacin (0.01 mg/mL, Fresenius Kabi, Bad Homburg, Germany). The number of wells was dependent on the size of the tissue. Cells were incubated in a humidified atmosphere at 37 °C and 5% CO_2_. The media was changed three times a week. Cells were harvested at a confluency of 80–90% but were cultured for at least 7 days with a maximum of 14 days. For long-term storage, 400,000 cells/vial were frozen in 1 mL freezing media (KSFM media, 25 mg BPE, 10% DMSO) and stored in liquid nitrogen.

Tissues for isolating cells after a forceps and cryo biopsy were taken ex vivo from a tracheal section of the same donor for direct comparison with AECs isolated using the conventional method (Fig. 1A). Briefly, the piece of tissue was placed on a dish and fixed using forceps. The forceps and cryo biopsy (Fig. 1A) was then taken tangentially from the tracheal mucosal tissue. For forceps biopsies, EndoJaw SwingJaw FB-231D (1.9 mm, Olympus, Hamburg, Germany) forceps were used, while an Erebecryo 2 with a flexible cryoprobe (1.7 mm, Erbe, Tübingen, Germany) was used for the cryo biopsy. For both biopsy techniques, epithelial cells were isolated as described above. However, instead of the scraping steps used for the conventional method, the biopsy pieces were incubated for 1 h in protease XIV (Sigma Aldrich) and the reaction was stopped by adding 2 mL 1x DPBS.

### ALI culture and HDM stimulation

One frozen vial with 400,000 cells of each sample was quickly thawed in a water bath at 37 °C and transferred to a coated (5 µg/mL fibronectin, Sigma Aldrich; 0.03 mg/ml PureCol, Advances BioMatrix; 1 mg/mL BSA, Sigma Aldrich) T75 flask in 10 mL KSFM media (Fig. 1B) after one washing step with 10 mL 1x DPBS and 8 min centrifugation (400 g, RT). Cells were cultivated at 37 °C and 5% CO_2_. During cell expansion, the media was changed three times a week and cells were cultured until a confluency of 80–90% was reached (approx. 8 days). Cells were then detached with soft trypsin (0.3 mg/mL trypsin, PAN-Biotech; 0.1 mg/mL EDTA, PAN-Biotech; 1 mg/mL glucose, VWR, Radnor, United States; 2 U/mL P/S, Gibco), counted, and seeded onto coated (5 µg/mL fibronectin, Sigma Aldrich; 0.03 mg/ml PureCol, Advances BioMatrix; 1 mg/ml BSA, Sigma Aldrich) 12-well cell culture inserts (12 mm diameter, 12-well plate size, 0.4 μm pore size, PET membrane transparent, VWR) with a cell density of 40,000 cells per well. The cells were seeded in media containing a 1:1-mixture of bronchial epithelial cell media (BEpiCM, ScienCell, Carlsbad, United States) and Dulbecco’s modified eagles’ media (DMEM with 4500 mg/mL D-glucose, StemCell, Vancouver, Canada), supplemented with bronchial epithelial cell growth supplement (BEpiCGS, ScienCell), 10 U/mL P/S, and 16 ng/mL EC-23 (Tocris, Bristol, United Kingdom, referred to as ALI-media). On the basal side of each insert, cells were kept in 1.5 mL ALI-media while apically cells were cultured in 0.5 mL ALI-media. During days 7–10, transepithelial electrical resistance (TEER, Millicell ERS-2 Epithelial Volt-Ohm Meter, Merck, Darmstadt, Germany) was measured. When a value of 224 Ω/cm^2^ was reached, the apical media was removed and cells were exposed to air. Cells were harvested at different time-points (1 week, 2 weeks, or 3 weeks) after air exposure. After 3 weeks, some fully-differentiated AECs were additionally treated with 10 µg/mL HDM (Mite Dust *D. preronyssinus*, Greer, Baar, Switzerland) for 6–24 h to perform functional analyses in response to the allergen exposure.

### Flow cytometry

Flow cytometry was performed after submerged culturing (day 8) and during ALI when AECs were first exposed to air as well as after 1, 2 or 3 weeks. AECs were harvested at each time-point. AECs were first washed with 1x DPBS, incubated with 1 mL calcium starvation media (S-MEM, Gibco) for 30 min at 37 °C, and then detached with 0.5 mL soft trypsin for 10 min at 37 °C. Residual adherent cells were scraped off the membrane using a cell scraper.

The staining of AECs for flow cytometry was performed as previously described [18]. The following antibodies were used to investigate distinct differentiation stages of epithelial cells: CD45 (protein tyrosine phosphatase receptor type C, PTPRC) for leukocytes (1:400, BV650, Cat#368535, Biolegend, San Diego, United States), CD326 (epithelial cell adhesion molecule, Ep-CAM) for epithelial cells (1:200, PerPCy 5.5, Cat# 324214, Biolegend), CD49f (integrin α6) for basal cells (1:200, BV421, Cat#313623, Biolegend), CD271 (nerve growth factor receptor, NGFR) for basal cells (1:400, PECy7, Cat#345110, Biolegend), CD66a/c/e (carcinoembryonic antigen-related cell adhesion molecule 1/6/5, (CEACAM1/6/5) for club cells (1:200, BV605, Cat#342324, Biolegend), tetraspanin-8 (TSPAN8) for goblet cells (1:200, APC, Cat# 363706, Biolegend), and acetylated α-tubulin (ac. α-tubulin) for ciliated cells (1:400, AF546, Cat#sc-23950, Santa Cruz). After antibody incubation, cells were fixated with 2% paraformaldehyde (PFA, Sigma Aldrich Aldrich) and measured on a CytoflexLX (Beckmann Coulter, Brea, United States). The detailed gating strategy is shown in the supplementary Fig. 1, which was based on Bonser et al. [19]. The software tool FlowJo (Version 10.8.1, BD, Franclin Lakes, United States) was used to analyze the data.

### Immunofluorescence staining and periodic acid-Schiff (PAS) reaction

AECs were harvested after 1, 2 or 3 weeks in ALI. First, the membrane of each well was rinsed with 0.5 mL 1x DPBS and cut out with a scalpel. For immunofluorescence staining, two-thirds of one insert membrane was fixated in ice-cold methanol (99.8%, Sigma Aldrich) and incubated at − 20 °C for 20 min followed by a 10 min incubation at RT and a washing step in 1 mL 1x DPBS. The membrane was then incubated in 0.5 mL washing buffer [Triton-X100 (0,1% v/v, Sigma Aldrich) in 1x DPBS] for 5 min at RT. This washing step was repeated a total of three times followed by a blocking step with 3% BSA for 1 h and an overnight incubation with the primary antibody monoclonal mouse anti-zonula occludens 1 (ZO-1; 1:100, Cat# 33-9100, Invitrogen, Waltham, United States) at 4 °C. The next day, the antibody solution was removed and the membrane was incubated in 0.5 mL washing buffer for 5 min at RT. This washing step was repeated a total of three times. Cells were then incubated with the secondary antibody Alexa Fluor 555 anti-mouse IgG (1:500, Cat#A32727, Invitrogen) for 1 h at 37 °C and 5% CO_2_. The antibody solution was removed and the membrane was washed with 0.5 mL washing buffer for 5 min. This washing step was repeated a total of three times. The cells were then stained apically with the following primary antibodies: monoclonal rabbit anti-mucin-5AC (MUC5AC; 1:400, Cat #61193, Cell Signaling, Danvers, United States) for the identification of goblet cells or monoclonal rabbit anti-acetylated α-tubulin (ac. α-tubulin, 1:800, Cat#5335, Cell Signaling) for ciliated cells. After removal of the antibody solution, the membranes were washed again with 0.5 mL washing buffer for 5 min, which was repeated a total of three times. Cells were then incubated with secondary antibodies Alexa Fluor 488 anti-rabbit IgG (1:500, Cat# A11008, Invitrogen) for 1 h at 37 °C and 5% CO_2_. Cell nuclei were counterstained with Hoechst 3342 Solution 15 (0.5 µg/mL, Chemometec, Lillerød, Denmark) before the membranes were covered with fluorescence mounting media (Dako Omnis, São Paulo, Brazil) and cover slips (Engelbrecht GmbH, Edermünde, Germany).

Quantitative analyses were performed based on images using the Echo Revolve (BICO, San Diego, United States) microscope. For each donor, a total of 10 images per condition were taken across the stained membrane; MUC5AC^+^ or ac. α-tubulin^+^ cells were additionally counted using the image analysis software tool Cellprofiler (Version 4.2.6, Cellprofiler, Cambridge, United States). Mean values were calculated for each donor and condition which were then related to the observed membrane surface measured in cm^2^. Representative images of the immunofluorescence staining were taken with an Axio Observer Z1 (Zeiss, Oberkochen, Germany) microscope.

As previously described for PAS staining [20], a part of the membrane was fixated in 4% PFA, dehydrated by an ascending ethanol series and xylene as intermedium (HistoCore PEARL, Leica Biosystem, Wetzlar, Germany), and embedded in paraffin (TES 99, Medite, Burgdorf, Germany). Using a microtome (Microm HM 340E, Thermo Scientific, Waltham, United States), 3 μm-thick sections were cut. Images were taken with an Echo Revolve (BICO) microscope.

### Scanning electron microscope (SEM)

AECs for SEM were harvested at each time-point after 1, 2 or 3 weeks in ALI. Briefly, cells were washed with 0.5 mL 1x DPBS, then the membrane was cut out with a scalpel. For SEM, cells were fixated in 3% (v/v) glutaraldehyde (Agar scientific, Stansted, United Kingdom), rinsed with 0.1 M sodium phosphate buffer (Merck) and dehydrated in an ascending ethanol series [20]. Samples were then subjected to critical point drying in liquid CO_2_ and coated with a 10 nm gold/palladium layer (Sputter Coater EM SCD500, Leica). SEM was performed in a high vacuum environment with environmental scanning electron microscopes (XL30 ESEM FEG, Philips or Quattro S ESEM, Thermo Scientific) and 10 kV acceleration voltage. To evaluate ciliation, SEM analysis was conducted by the core facility for electron microscopy of the medical faculty of Rheinisch-Westfaelische Technische Hochschule (RWTH) Aachen University.

### Statistical analysis

Statistical analyses were performed with GraphPad Prism (v9.4.1). Results were analyzed using two-way analysis of variance with Tukey’s *post-hoc* test. A p-value of *p* < 0.05 was considered statistically significant.

### Proteomics analysis

For the analysis of the proteome, material from the same subjects (*n* = 3) was used as in the other outcomes. AECs were lysed in urea buffer (7 M urea, 2 M thiourea, 0.1% sodium deoxycholate, 30 mM TRIS, pH 8.5) directly from the membranes and aliquots of 10 µg were processed according to the SP3 protocol [14] with minor modifications. Briefly, samples were reduced with 20 mM dithiothreitol (DTT, 30 min, RT), alkylated with 2-iodoacetamide (50 mM, 30 min, RT, in the dark) and quenched with 50 mM DTT. Subsequently, 100 µg carboxylate-modified magnetic beads and acetonitrile (70% final concentration) were added to the samples and the mixture was incubated for 20 min. The beads then were washed two times with 70% ethanol, once with acetonitrile and digested over night with 0.2 µg trypsin in 20 µL 50 mM ammonium bicarbonate. Samples were dried and resuspended in 0.1% trifluoroacetic acid and 300 ng of each were analyzed on a Vanquish Neo UHPLC coupled to an Orbitrap 480 mass spectrometer (both Thermo Scientific) with 0.1% formic acid (FA) as mobile phase A and mobile phase B being 80% ACN and 0.1% FA. Peptides were preconcentrated on an Acclaim PepMap 100 trap column (100 μm x 2 cm, Thermo Scientific) and Peptide separation was performed using a DNV PepMap Neo separation column (75 μm x 150 mm, Thermo Scientific) with a gradient from 1% B to 40% B within 120 min at a flow rate of 400 nL/min at 60 °C. The MS1 scan range was set to 350–1450 m/z with a resolution of 120,000, a normalized AGC target of 300% and a maximum injection time (MIT) of 54 ms. MS2 scans were acquired with a normalized collision energy of 30%, a resolution of 30,000, and a scan range of 145–1450 m/z with a normalized AGC target of 2500% and a MIT of 80 ms. In total,40 isolation windows between 350 and 1450 m/z were cycled through, whereas one MS1 scan was recorded every 21 MS2 scans. Protein quantification was done with DIA-NN [21] using the library free mode with the SwissProt database of Homo sapiens (ver. 2023_02). The report file was filtered for q-values < = 0.01, samples were normalized using the cyclic loess method from the R limma package and the protein quantities were calculated using the MaxLFQ algorithm of the DIA-NN R package. Differences between groups were calculated by repeated measures ANOVA with Tukey HSD post-hoc test. To correct for multiple testing, the Benjamini-Hochberg method was used for the ANOVA p-value.

## Results

### Vitality, yield, and purity of AECs

**Fig. 2:**
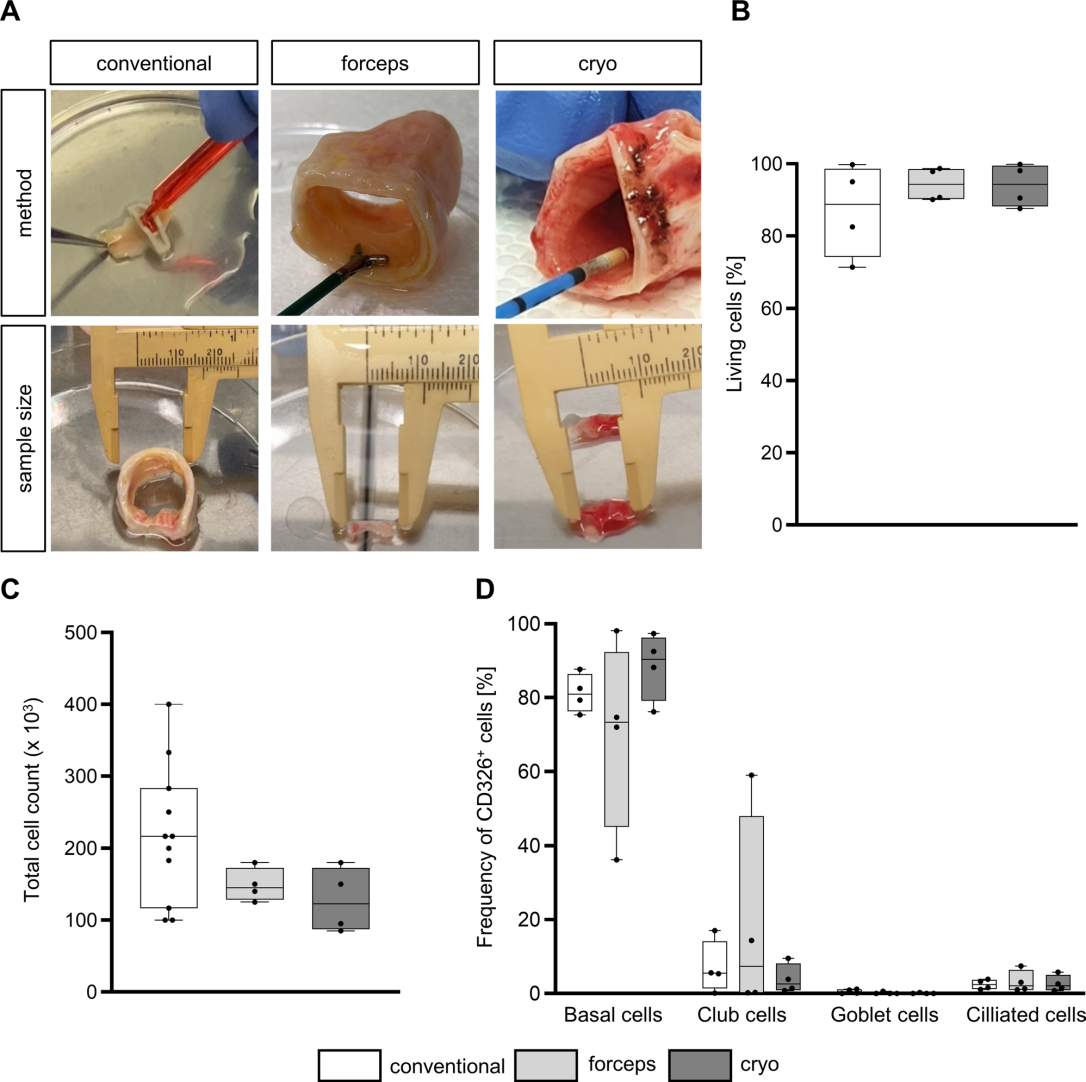
Sampling of primary airway epithelial cells (AECs): vitality, yield, and purity. **(A)** Sampling method and tissue size of the conventional method or by forceps and cryo biopsy. In the conventional method, AECs were scraped off the mucosal surface after enzymatic digestion. Forceps and cryo biopsy were taken tangentially followed by enzymatic digestion. Representative pictures of tissues sizes used for cell isolation are shown for each sampling procedure. **(B)** Vitality directly after scraping or biopsy. Results are shown as box and whiskers (plot: min to max, show all points, *n* = 4). **(C)** Cell yield (day 8) of each sampling method. Results are shown as box and whiskers (plot: min to max, show all points, conventional: *n* = 11, forceps: *n* = 4, cryo: *n* = 4). **(D)** Flow cytometry was performed to determine cellular composition using the following markers for each cell type of AECs (CD326^+^): percentage of basal (CD49f^+^CD271^+^), club (CD66a/c/e^+^), goblet (TSPAN8^+^), and ciliated cells (ac. α-tubulin^+^). Results are shown as mean ± standard deviation. All results were analyzed by two-way analysis of variance with Tukey’s post-hoc test

Primary AECs were isolated ex vivo from healthy tracheal transplant tissues comparing conventional sampling method to forceps and cryo biopsy. As expected, tissue size differed between each of the methods (Fig. 2A) which were generally smaller for biopsy samples in comparison to the conventional method. After the isolation of AECs, cell viability was equivalent for each of the sampling techniques (Fig. 2B). After the expansion phase, harvested cells showed no significant difference in cell yield, respectively (Fig. 2C). Cell composition after the basal cell expansion phase (day 8) was examined by flow cytometry (Fig. 2D, gating strategy: supplementary Fig. 1) discriminating between basal, club, goblet, and ciliated cells. As to be anticipated at this stage, the proportion of basal cells (CD49f^+^CD271^+^) was 70–88%. The remaining cells were mostly club cells (CD66a/c/e^+^), with the largest proportion in the forceps biopsy group. Overall, there was no significant differences in yield, vitality, and cellular composition related to the sampling method.

### Differentiation of AEC-ALIs

**Fig. 3:**
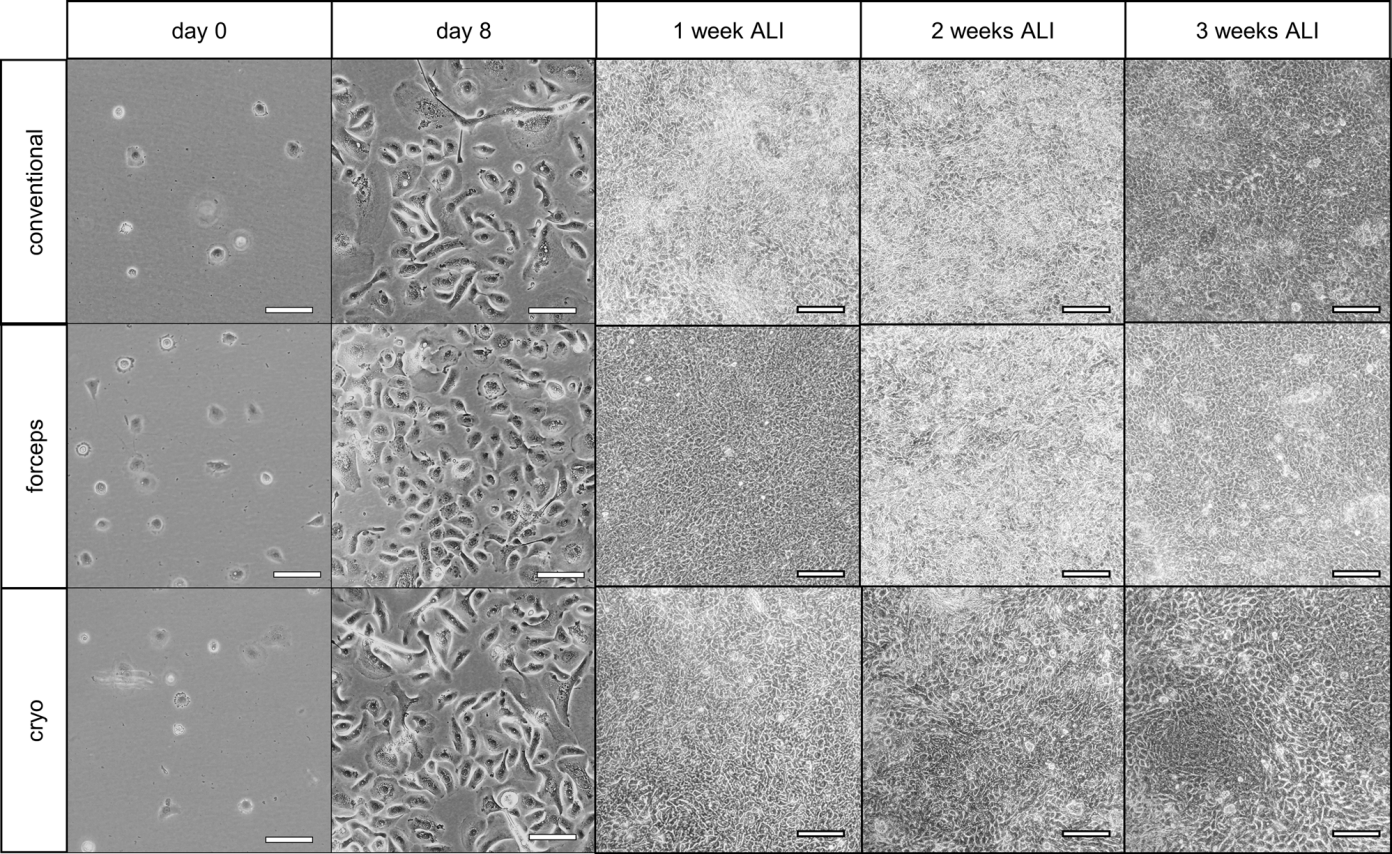
Cell morphology before and after air exposure. Cell morphology after thawing (day 0). After the expansion phase (day 8), cells were approximately 80–90% confluent and were transferred to the inserts. The morphology of airway epithelial cells during cultivation after 1, 2, and 3 weeks in ALI. Scale bar 100 μm

The cell morphology and composition of AECs at each differentiation stage during 3 weeks in ALI depending on the sampling method was examined using various experimental techniques. After thawing (day 0), basal cells reached a confluence of 80–90% after 8 days (Fig. 3). When AECs were exposed to air for 1 week, characteristic cobblestone-like structure AECs were already visible, independent of the sampling method which persisted over the 3-week cultivation period. Development of cilia is one critical characteristic feature of differentiated AECs; this was analyzed by SEM after 3 weeks in ALI.

**Fig. 4:**
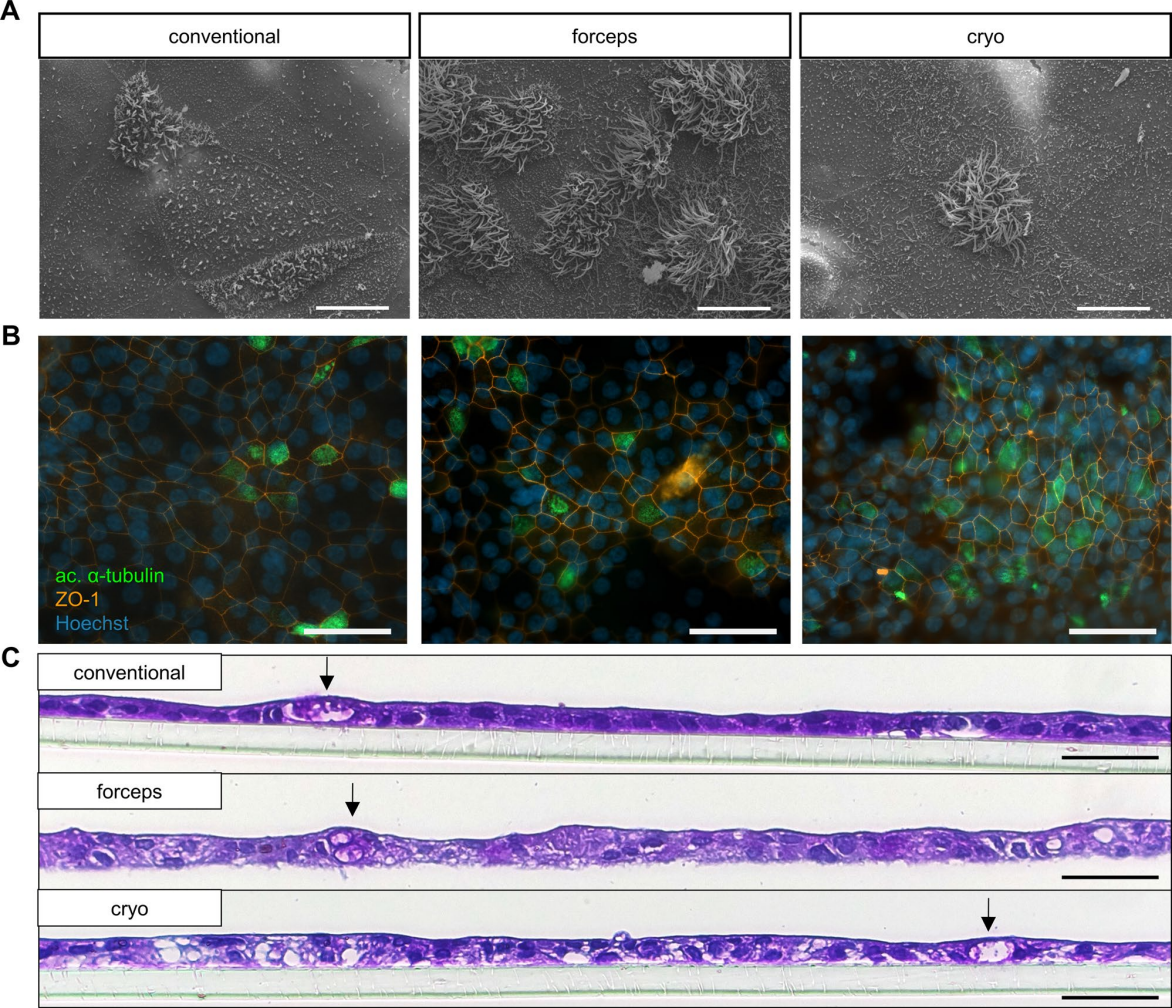
Differentiation of airway epithelial cells (AECs) in air-liquid interface (ALI). **(A)** Ciliation was analyzed by scanning electron microscopy after 3 weeks in ALI using conventional, forceps, and cryo-sampled AECs (*n* = 5); representative pictures are shown; scale bar 10 μm. **(B)** Representative pictures of ciliation were analyzed by immunofluorescence staining (green–ac. α-tubulin; blue–Hoechst, red–ZO1) after 3 weeks in ALI using conventional, forceps, and cryo-sampled AECs (*n* = 5; scale bar 40 μm). **(C)** Differentiation of ALI-AECs after 3 weeks using conventional, forceps, and cryo-sampled cells analyzed by *periodic acid-Schiff* reaction (PAS, *n* = 5); goblet cells are marked by an arrow; representative pictures are shown; scale bar 50 μm

Comparable ciliation was detected in ALI-AECs after each of the sampling procedures (Fig. 4A). This was also confirmed by immunofluorescence staining with ac. α-tubulin, tight junctions (ZO1), and nuclei (Hoechst 3342) (Fig. 4B). As identified by histological staining via PAS reaction, the number of goblet cells was similar regardless of the sampling method (labelled by arrows, Fig. 4C).

**Fig. 5:**
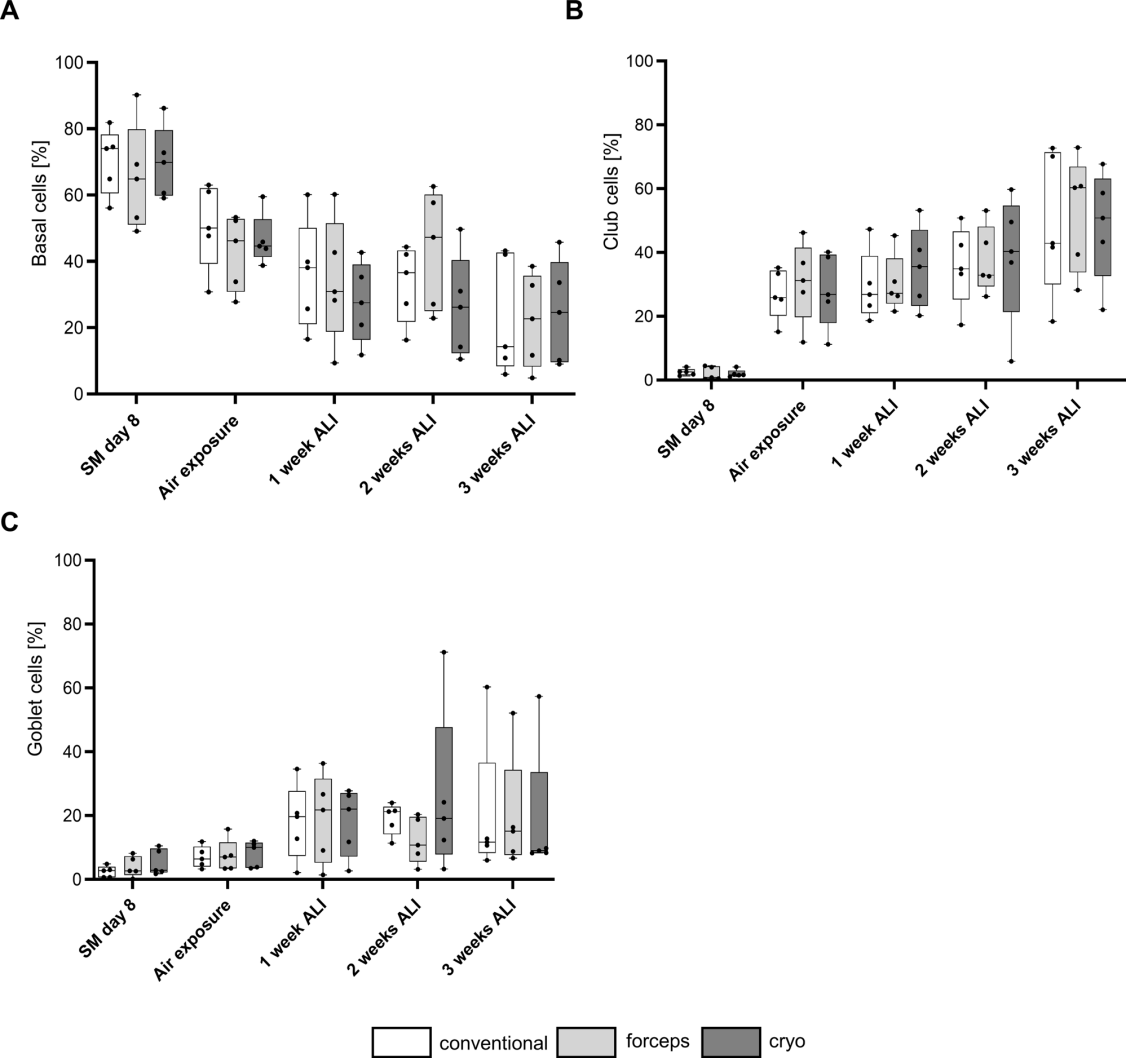
Differentiation of airway epithelial cells (AECs) in air-liquid interface (ALI). Cellular composition of cultivated AECs on day 8, at air exposure, and after 1, 2, or 3 weeks in ALI was analyzed by flow cytometry (*n* = 5) using the following markers for each epithelial cell type (CD326^+^): **(A)** percentage of basal (CD49f^+^ CD271^+^), **(B)** club (CD66a/c/e^+^), **(C)** goblet (TSPAN8^+^). Results are expressed as mean ± standard deviation (plot: min to max, show all points). Statistical analyses were performed by two-way analysis of variance with Tukey’s post-hoc test

Cell composition during each of the differentiation steps was analyzed using flow cytometry (Fig. 5A), confirming the comparability of each of the applied sampling methods. After the expansion phase, the majority of the cells were classified as basal cells. The proportion of basal cells decreased over time when cells were exposed to air and cultured for 1, 2, or 3 weeks in ALI. Conversely, the proportion of goblet cells progressively increased, reaching a maximum of ≈20% after 3 weeks in ALI (Fig. 5C). The proportion of club cells also increased from < 5–30% after air exposure which further increased to > 40% during the differentiation phase in ALI (Fig. 5B, further significances over differentiation time see supplementary Fig. 3). Hence, the sampling method had no impact on ciliation, goblet cell differentiation, and cellular composition over the entire cultivation period.

### Response of ALI-AECs to HDM stimulation

**Fig. 6:**
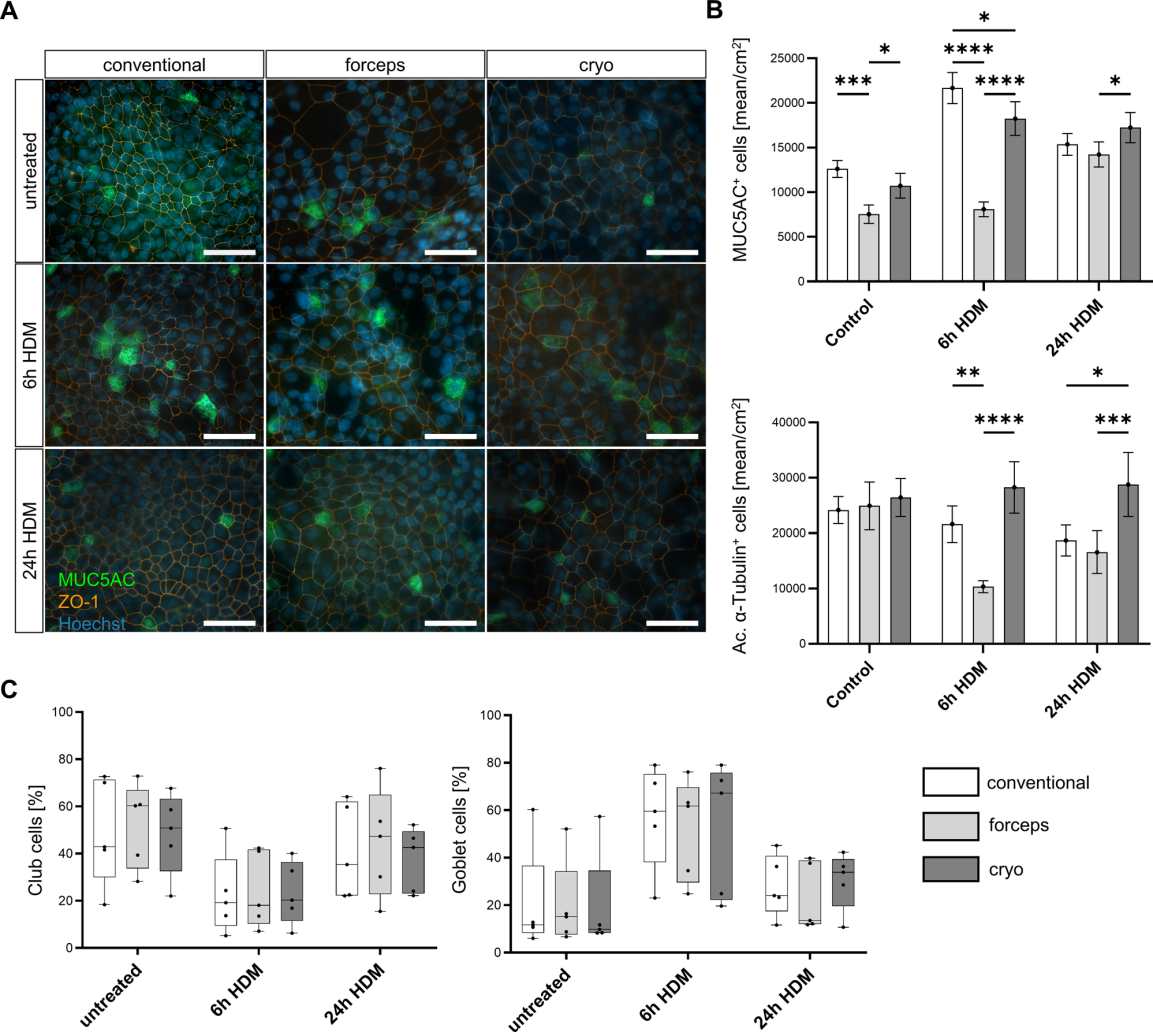
Influence of house dust mite extract (HDM) on differentiation of airway epithelial cells (AECs). After 3 weeks in ALI, AECs from conventional, forceps, and cryo-sampled cells were untreated or stimulated with HDM for 6–24 h (*n* = 4–5). **(A)** Immunofluorescence staining of goblet cells (green–MUC5AC; blue–Hoechst, red–ZO1) (*n* = 5; scale bar 40 μm); representative pictures are shown. **(B)** Numbers by amount of MUC5AC^+^ goblet cell or ac. α-tubulin^+^ cells were determined per cm^2^ (*n* = 4–5). (**C)**. Analyses of goblet cells (TSPAN8^+^) and club cells (CD66a/c/e^+^) by flow cytometry (*n* = 5). (**B) + (C)** Results are expressed as mean ± standard deviation (plot: min to max, show all points). Statistical analyses were performed by two-way analysis of variance with Tukey’s post-hoc test, *p < 0.05; **p < 0.01; ***p < 0.001; ****p < 0.0001

To evaluate the impact of a clinically-relevant allergen on differentiated ALI-AECs obtained from distinct sampling methods, cells were exposed to HDM for 6–24 h after 3 weeks of ALI culture (*n* = 5, Fig. 6A and B). Immunofluorescence staining was performed to detect nuclei (Hoechst^+^), goblet cells (MUC5AC^+^), and tight junctions (ZO1^+^) of AECs. In untreated ALI-AECs, significantly fewer MUC5AC^+^ cells were detected in samples obtained using forceps biopsies compared with the conventional method. The number of MUC5AC^+^ cells was lower in cells from forceps versus cryo biopsies. In addition, the number of MUC5AC^+^ cells was lower after 6 h HDM stimulation in the forceps and cryo-sampled cultures compared with the conventional method. Cryo-sampled ALI-AECs showed more MUC5AC^+^ cells than ALI-AECs from forceps biopsies. After 24 h treatment with HDM, there was a significant increase in the number of MUC5AC^+^ cells in ALI-ACEs from cryo vs. forceps biopsies, but no significant difference between the biopsies and conventional methods (further significances regarding stimulation see supplementary Fig. 4A).

The magnitude of ciliation in response to HDM was measured by immunofluorescence staining (Fig. 6B). In untreated cells, the number of ciliated cells did not differ between each sampling method. After 6 h HDM stimulation, significantly fewer ac. α-tubulin^+^ cells were identified in the forceps-sampled ALI-AECs compared with the conventional and cryo-sampled cultures. The highest ciliation was observed after 24 h HDM in ALI-AECs obtained from cryo biopsies, with significant differences versus ALI-AECs obtained using the conventional method or forceps biopsies.

The effect of HDM on goblet and club cells was also analyzed using flow cytometry (Fig. 6C). The relative number of goblet cells (TSPAN8^+^) was comparable between each sampling method in untreated cells and after 6–24 h stimulation with HDM. The relative number of club cells in response to HDM did not differ significantly between the three sampling methods (Fig. 6C). There was a trend towards a short-term increase in goblet cells and a lower percentage of club cells after 6 h HDM stimulation. However, this effect was lost in AECs exposed to HDM for 24 h. These data indicate that the tissue sampling method, mainly by forceps biopsy, may affect the response to extrinsic allergen stimuli.

### AEC proteome analyses

**Fig. 7:**
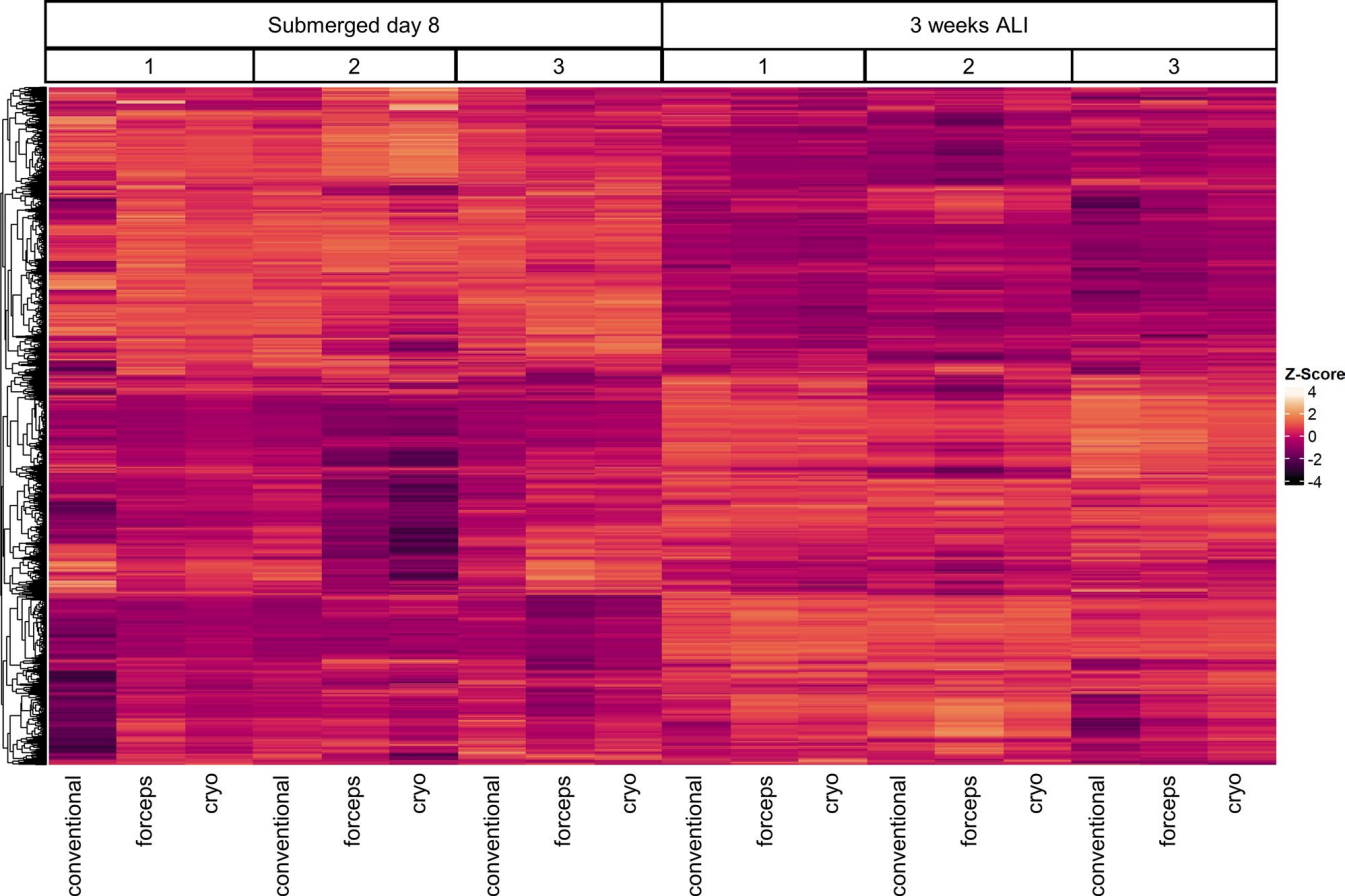
Influence of the sampling method on the proteome. Protein expression patterns of airway epithelial cells (AECs) after 8 days of expansion of basal cells and after 3 weeks in ALI of each sample are shown as a heat map. The three different sampling methods (conventional, forceps, and cryo) were compared, and three healthy subjects were analyzed

To evaluate the impact of different sampling methods on both cellular behaviour and global protein expression patterns in AECs, untargeted proteome analyses were performed in AECs from three subjects at two different time-points (basal cells after the expansion phase on day 8 and after 3 weeks in ALI, Fig. 7). Overall, more than 8,500 proteins were detected, with no significant difference between sampling groups (Fig. 7 and supplementary Fig. 5). The supplementary material provides an overview of the top 20 regulated proteins from the respective sampling method (supplementary Tables 1 and 2). As expected, a higher variance of expressed proteins was identified when comparing basal cells to ALI-AECs after 3 weeks in ALI (Fig. 7). These differences were even more pronounced when the two cell subgroups within a donor were directly compared (Fig. 7). Although changes of the proteome were seen based on the differentiation stage and donor, the sampling method of AECs did not have a significant impact on the proteome.

## Discussion

There is a strong need for adequate model systems that can help to enhance our understanding of complex airway diseases while reducing the use of animals. The availability of primary donor tissue remains the most challenging aspect of primary human respiratory cell culture models. To overcome this limitation, we aimed to delineate an alternative method to isolate primary AECs from the lower respiratory tract. To the best of our knowledge, this is the first study of its kind showing that the cell yield, morphology, vitality, differentiation capacity, and the proteome of AECs are equivalent after conventional isolation [22] in direct comparison to isolated and differentiated ALI-AECs cells from forceps and cryo biopsies. This is an important finding because it opens up new avenues to perform mechanistic studies on AECs not only in patients with end-stage lung disease. Treatment with HDM led to minor differences in mucociliary differentiation between isolation method groups, but not in the frequency of goblet or club cells.

In 2020, the European Union and Norway used over 8 million animals for research purposes which were mainly used for basic, translational, and applied research (5.8 million) [23]. Most animal models are not able to fully reflect human-specific features and are therefore unable to reproduce the complex pathophysiological mechanisms that lead to diseases such as asthma and are of poor predictive value for humans [24]. For this reason, in vitro cell culture models using human primary cells are becoming increasingly important as they offer several advantages over experiments involving animals. Cell culture models allow isolated cells to be exposed to disease-relevant stimuli, offering the ability to study cell functions of healthy and diseased individuals under controlled conditions. Human primary AECs are predominantly obtained from patients with a severe lung condition (such as emphysema, cystic fibrosis, or pulmonary fibrosis) who are undergoing lung resection, transplantation, or autopsy. These invasive procedures limit access to ALI-AECs for research purposes [25]. With our study results, we present a new tissue sampling alternative to isolate AECs also from the lower airways while applying a procedure which is commonly used for diagnostic while performing a bronchoscopy. Distal airway samples (bronchial or tracheobronchial AECs) are widely used for research [25], while nasal epithelial cells from nasal brushings is a less invasive source [25, 26], but with the caveat of a lower cell yield and a higher risk of contamination [27, 28]. With cryo and forceps biopsies, we have confirmed that adequate numbers of viable AECs for analyses in ALI culture can be isolated. Although nasal brushings are a valuable alternative due to the overlapping characteristics of epithelial cells from the upper and lower respiratory tract [20], these provide limited information on the behaviour of epithelial cells from the lung and bronchi. Thus, tracheal and bronchial AECs are a more representative model for diseases such as COPD or asthma affecting the lower respiratory tract [29]. Since our data confirms that forceps and cryo biopsies are a viable alternative to the conventional method, future mechanistic experiments can now be initiated at an earlier-disease stage.

Studies using human bronchial AECs derived from forceps biopsies have investigated epithelial inflammation in patients with asthma or chronic bronchitis [30, 31]. However, our study is the first to characterize the effect of forceps or cryo biopsy on the isolation and functional properties of primary AECs. As expected, the tissue size varied markedly between the conventional and both biopsy methods. After normalization of cell yield to the cell growth surface area, no significant differences between the conventional method and the biopsies were detected demonstrating a comparable proliferation behaviour and capacity.

Cryotherapy has previously been used for various tumours, and this causes cell death and tissue necrosis [32]. In contrast to cryotherapy and cryo-biopsies in clinical practice, the freezing time during cryo biopsy in the present study was restricted to one second. Using a short freezing time, we increased cell viability to obtain cells from bronchial mucosa and to potentially reduce the risk of bleeding and pneumothoraxes for future clinical application of the technique. Furthermore, cryo biopsy is the preferred method for pathological examinations in adults due to its lower risk and higher success rates [33]. This contributed to our observation that no significant difference was present for any of the analyzed sampling procedures including cell yield, vitality, morphology, and differentiation. Similarly, cell morphology remained unaffected during the expansion phase with the characteristic oval shape during ALI. We showed that the differentiation capacity and ciliation of isolated cells was comparable between the sampling methods used, with a progressive shift from basal to goblet or club cells that was greater when air exposure was longer. It has been postulated that the differentiation capacity can vary depending on the sampling site, passage, and ALI protocol. Our results showed that the quality and cell behaviour of isolated cells were highly comparable for all three sampling methods. Thus, each provides a valuable tool to isolate cells to be differentiated to fully functional AECs.

In addition to analyzing the impact of sampling methods, we investigated in vitro responses to external allergen exposure. In the lung, the respiratory epithelium reacts to HDM with remodelling processes, including loss of epithelial integrity, thickening of the basement membrane, mucus-gland and goblet-cell hyperplasia, smooth muscle hyperplasia, and increased airway vascularity [34, 35]. In addition, the HDM allergen contains proteases that directly affect the airway epithelium [36]. Despite less goblet cells in unstimulated ALI-AECs from biopsies compared with the conventional technique, HDM stimulation induced goblet cell production in all three sampling groups. MUC5AC, a ‘response mucin’ secreted after allergen exposure [37, 38], was found to be increased in goblet cells at 6 and 24 h after HDM treatment, supporting the development of goblet cell metaplasia. Not only is the *MUC5AC* gene strongly upregulated in secretory cells of the respiratory tract, the cell surface glycoprotein TSPAN8 is also co-expressed in 60% of ALI-AECs [39]. In response to external stimuli, TSPAN8 is downregulated and regulates mucin secretion by controlling the number of mucin granules [39]. In our ALI-ACEs, the HDM stimulus after 6 h elicited an increased goblet cell phenotype combined with induction of TSPAN8^+^ cells. However, levels were comparable to control cells at 24 h after HDM exposure. Overall, it was shown that ALI-ACEs derived from each of the sampling methods responded to HDM stimulation with elevated goblet cells, and had a significant effect on cell ciliation. This may be related to a shift towards epithelial mesenchymal transition, one of the processes associated with atopic diseases including asthma that can be triggered by allergen exposure [34, 40, 41]. The detection of ac. α-tubulin showed that HDM had no influence on the ciliation of cryo-sampled ALI-AECs whereas the expression of MUC5AC was elevated, resulting in goblet cell metaplasia. In summary, cells from biopsy cultures responded to HDM, but to varying extents.

As we anticipated, proteome analyses showed different protein expression patterns between the expansion and differentiated phase of AECs, indicating significant protein profile changes after air exposure [42]. The current study found no meaningful influence of the AECs sampling method in submerged cultures or ALI. Variances in protein expression profiles were more strongly influenced by the differentiation stage or donor-specific disparities. The low variance within ALI cultures highlights the reproducibility of the differentiation protocols, which can be applied to each of the evaluated sampling methods.

Limitations of the study include discrepancies between outcomes measuring MUC5AC^+^ or TSPAN8^+^ cells in goblet cells using two independent techniques, including immunofluorescence and flow cytometry. The immunofluorescence staining provides a mean value of MUC5AC^+^ cells per cm^2^ while flow cytometry normalizes TSPAN8 as goblet cell marker to the total amount of ACEs. Future research should examine both markers in both methods to analyze the effects of sampling methods on goblet cell differentiation. The sampling methods under study lead to slight differences on ciliation after HDM stimulation, which also needs to be analyzed in more detail. Molecular analyses, SEM images or proteomics of the HDM stimulated AECs may additionally provide more information on ciliation. It should be noted that the variance with each of the sampling techniques in response to HDM may be related to an unknown HDM allergy of the donors. Information about potential allergies of the donors are not available due to data protection reasons.

## Conclusion

This study showed the feasibility and comparability of AEC cultures derived from biopsies compared with a standardized conventional method. Functional analyses identified minor changes in mucociliary differentiation in response to allergen stimuli. Due to the number of taken methodological approaches including the analysis of the cellular composition and the whole proteome during each of the differentiation steps of ALI-AECs, we propose that tissue from biopsies are a viable alternative. Of note, the biopsy-based techniques provide a unique opportunity to overcome the shortcoming of donor tissue and to improve current knowledge about mechanisms relevant to disease progression starting at an earlier stage or in less severe lung disease that do not require surgical treatment. Both biopsy methods are a valuable source for the isolation of primary epithelial cells from the airways, making their use for future studies easier and allowing the reduction of unnecessary animal experiments in lung research.

## Electronic supplementary material

Below is the link to the electronic supplementary material.

Supplementary Material 1

## Data Availability

Proteomics data that support the findings of the study will be deposited upon acceptance in a public accessible archive.

## Abbreviations

ac.α-tubulin: Acetylated α-tubulin
ACE: Airway epithelial cells
ALI: Air-liquid interface
BEpiCGS: Bronchial epithelial cell growth supplement
BEpiCM: Bronchial epithelial cell medium
BPE: Bovine pituitary extract
BSA: Bovine serum albumin
CEACAM1/6/5: Carcinoembryonic antigen-related cell adhesion molecule 1/6/5
COPD: Chronic obstructive pulmonary disease
DMEM: Dulbecco’s modified eagles’s medium
DPBS: Dulbecco’s phosphate buffered saline
EGF: Epidermal growth factor
Ep-CAM: Epithelial cell adhesion molecule
FBS: Fetal bovine serum
HDM: House dust mite extract
KSFM: medium-Keratinozyten-SFM medium
MUC5AC: Mucine 5ac
NGFR: Nerve growth factor receptor
P/S: Penicilllin-streptomycin
PAS: Periodic acid-Schiff reaction
PFA: Paraformaldehyde
Protease XIV: Protease from *Streptomyces **griseus*
PTPRC: Protein tyrosine phosphatase receptor type C
SEM: Scanning electron microscope
SM: Submerged
S-MEM: Calcium starvation medium
TEER: Transepithelial electrical resistance
TSPAN8: Tetraspanin-8
ZO-1: Zonula occludens-1
