# Supplementary material for: Bronchoscopic biopsies - a novel source for primary airway epithelial cells in respiratory research
Source: Respir Res. 2024 Dec 24;25:439. doi: 10.1186/s12931-024-03060-1 (PMC11669235; doi:10.1186/s12931-024-03060-1)
Supplement: Supplementary file 1 — Supplementary Material 1 [file 12931_2024_3060_MOESM1_ESM.docx]

**SUPPLEMENTAL FIGURES AND TABLES**

**Bronchoscopic biopsies - a novel source for primary airway epithelial cells in respiratory research**

Kimberly Barbet^1*^, Mona S. Schmitz^1*^, Dirk Westhölter^1^, Markus Kamler^2^, Stephan Rütten^3^, Anja Lena Thiebes^4^, Barbara Sitek^5,6^, Malte Bayer^5,6^, Michaela Schedel^1,7^, Sebastian Reuter^1^, Kaid Darwiche^8^, Anja E. Luengen^1^, Christian Taube^1^

^1^Department of Pulmonary Medicine, University Medical Center Essen, Ruhrlandklinik, Essen, Germany

^2^Department of Thoracic and Cardiovascular Surgery, University Medical Center Essen, Essen, Germany

^3^Institute of Pathology, Electron Microscopy Facility, RWTH Aachen University Hospital, Aachen, Germany

^4^Department of Biohybrid and Medical Textiles (BioTex), AME - Institute of Applied Medical Engineering, Helmholtz Institute, RWTH Aachen University, Aachen, Germany

^5^Medical Proteom-Center (MPC) Medical Faculty, Ruhr-University Bochum, Bochum, Germany

^6^Department of Anesthesia, Intensive Care Medicine and Pain Therapy, University Hospital Knappschaftskrankenhaus Boch

um, Germany

^7^Department of Pulmonology, University Medical Center Essen, Essen, Germany

^8^Interventional Pulmonology, Department of Pulmonary Medicine, University Medical Center Essen, Ruhrlandklinik, Essen, Germany

*Shared first authorship, contributed equally

**
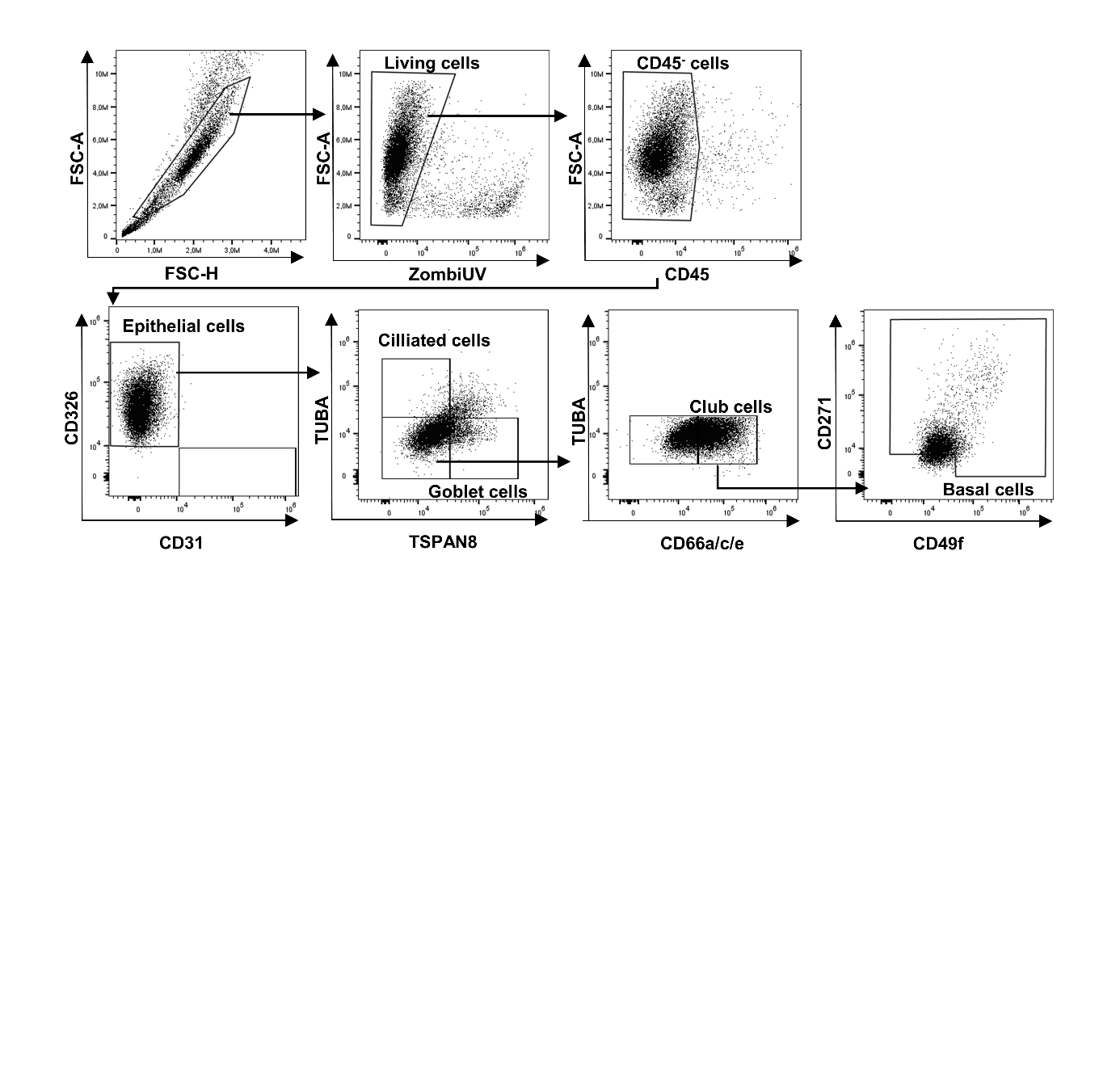
**

**Supplementary Figure 1.** **Gating strategy to discriminate subsets of primary epithelial cells.** Doublet exclusion was performed by plotting the height and width against the area via FSC-H/FSC-A and living cells were identified as ZombieUV^-^ cells. CD45^-^ cells were gated to exclude immune cells and CD326^+^CD31^-^ cells were defined as epithelial cells. The subpopulations of epithelial cells were distinguished using CD271^+^/CD49f^+^ for basal cells, CD66a/c/e^+^ for club cells, TSPAN8^+^ for goblet cells, and acetylated α-tubulin^+^ for ciliated cells. The gating strategy is adapted from Bonser et al, 2021 [3].

**
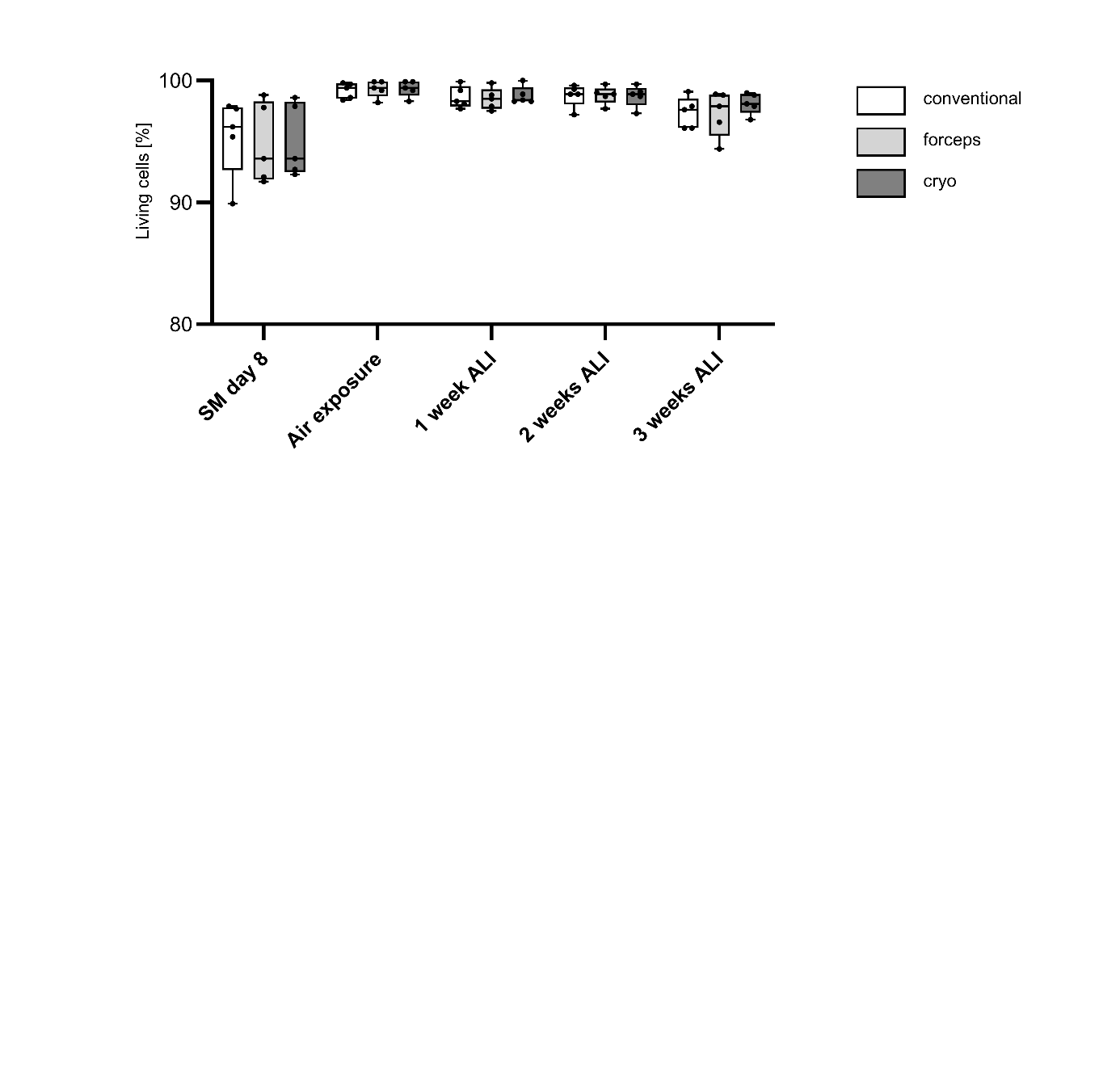
**

**Supplementary Figure 2. Viability during the differentiation of airway epithelial cells in air liquid interphase (ALI).** Living cells reflected as ZombieUV^-^ cells were shown at each of the time-points [submerged (SM) day 8, after air exposure, and after 1, 2, 3 weeks in ALI]. Results (n=5) are expressed as mean ± standard deviation (plot: min to max, show all points). Statistical analyses were performed by two-way analysis of variance with Tukey’s post-hoc test.

**
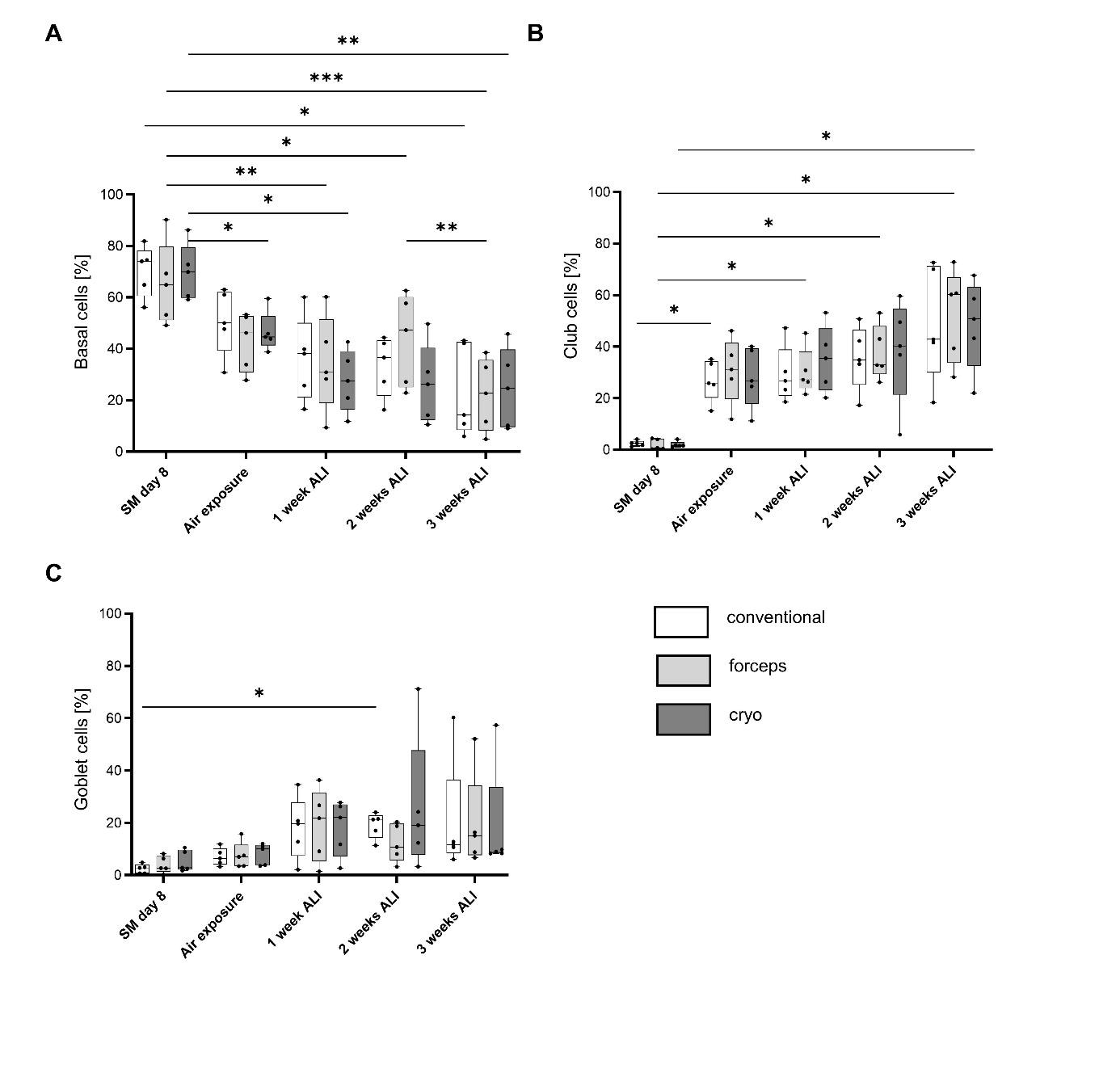
**

**Supplementary Figure 3. Cell composition of airway epithelial cells analysed by flow cytometry during differentiation [submerged (SM) day 8, at the time of air exposure, or differentiated after 1, 2, or 3 weeks in air-liquid interface (ALI)]. A)** The relative amount (%, n=5) of basal (CD271^+^/CD49f^+^) **B)** club (CD66a/c/e^+^) and **C)** goblet cells (TSPAN8). Results are expressed as mean ± standard deviation (plot: min to max, show all points. Statistical analyses were performed by two-way ANOVA with Tukey’s post-hoc test, *p<0.05; **p<0.01; ***p<0.001.

**
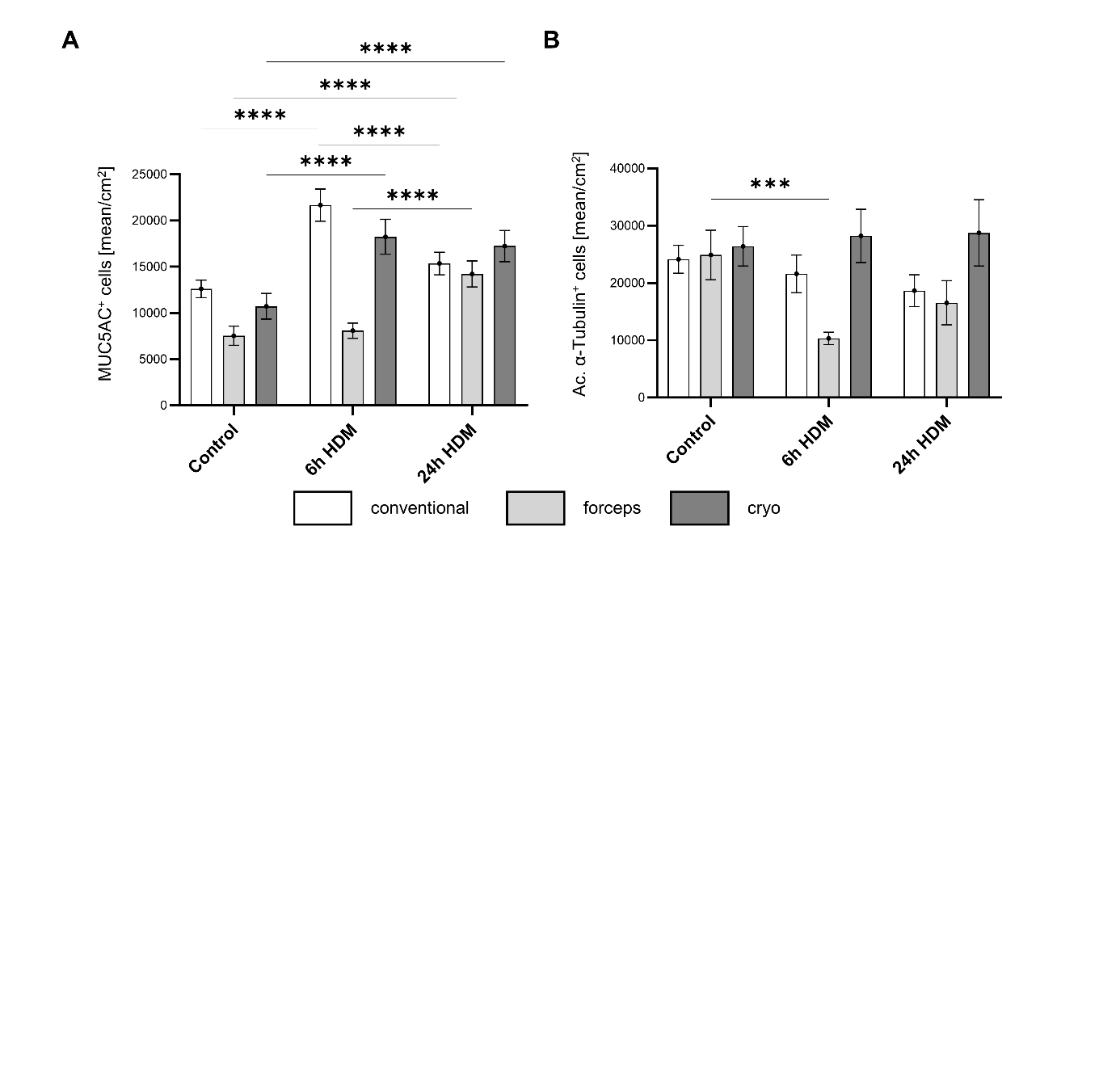
**

**Supplementary Figure 4. Influence of house dust mite (HDM) on the cell composition differentiation of airway epithelial cells (AECs).** After 3 weeks in air-liquid interface (ALI) from conventional, forceps, and cryo-sampled cells, AECs were untreated or stimulated with HDM for 6 h or 24 h (n=4-5,). **A)** Immunofluorescence staining of goblet cells (green–MUC5AC; blue–Hoechst, red–ZO1) (n=5; scale bar 40 µm); representative pictures are shown. **B)** Numbers by amount of MUC5AC^+^ goblet cells or ac. α-tubulin^+^ were determined per cm^2^ (n=4–5). Results are expressed as mean ± standard deviation following Gaussian error propagation. Results are expressed as mean ± standard deviation. Statistical analyses were performed by two-way analysis of variance with Tukey’s post-hoc test, ***p<0.001; ****p<0.0001.


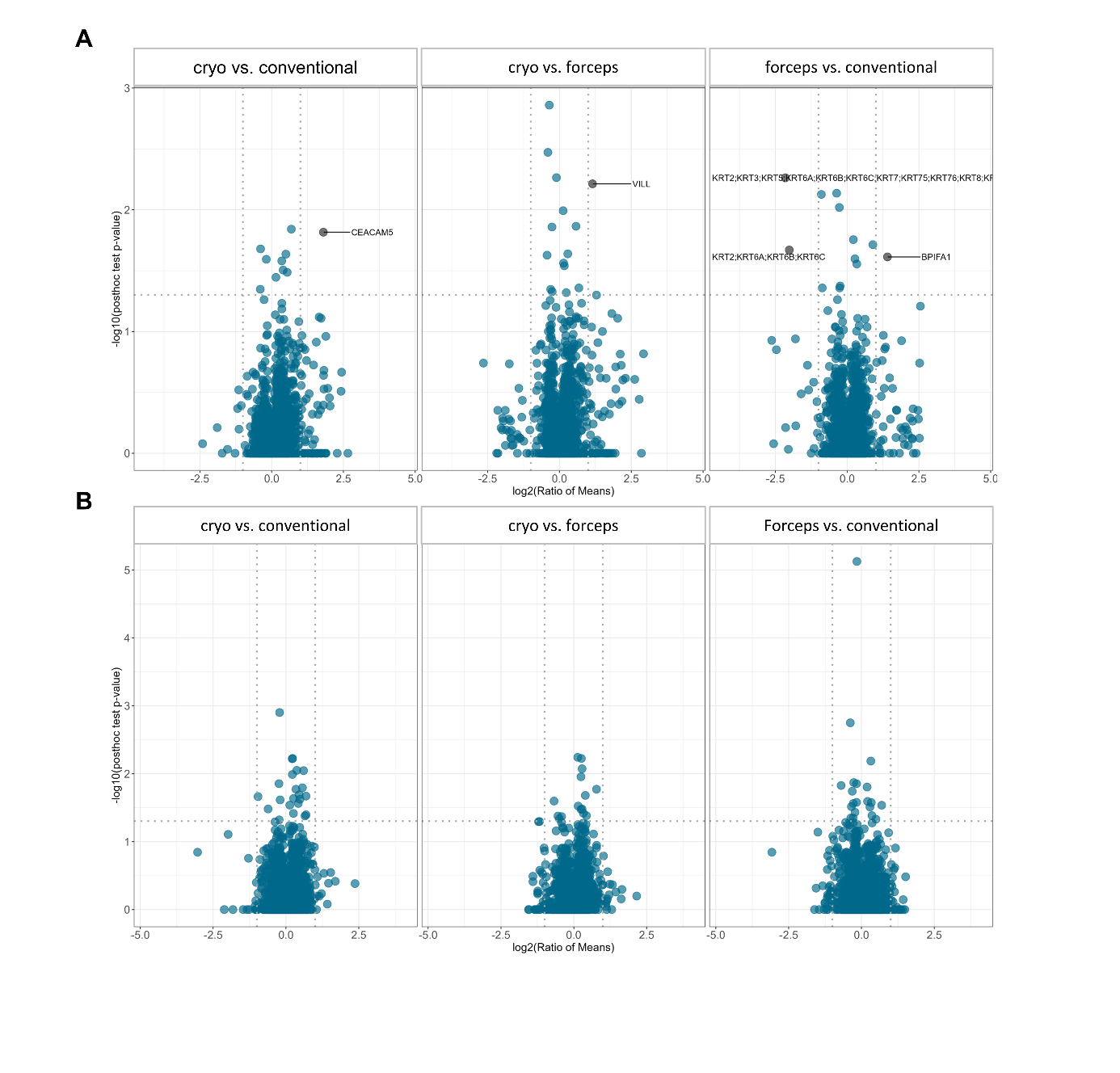


**Supplementary Figure 5. Influence of the sampling method on the proteome of airway epithelial cells (AECs).** AECs from the healthy donor lungs were obtained using different sampling methods (conventional, forceps, and cryo) and the proteome was analysed at two different time-points: **A)** after 8 days of expansion, and **B)** after 3 weeks in air-liquid interface.

**Supplementary Table 1: Top 20 regulated proteins (based on SwissProt) in basal cells after 8 days expansion.** Due to the use of MS/MS in some cases, the sequence could not be aligned to a single protein. Therefore, protein groups or gene groups are displayed.

**Supplementary Table 2: Top 20 regulated protein group after three weeks air liquid interface.** Due to the use of MS/MS in some cases, the sequence could not be aligned to a single protein. Therefore, protein groups or gene groups are displayed.
